# Rapid identification of melioidosis agent by an insulated isothermal PCR on a field–deployable device

**DOI:** 10.7717/peerj.9238

**Published:** 2020-05-27

**Authors:** Kek Heng Chua, E. Wei Tan, Hwa Chia Chai, SD Puthucheary, Ping Chin Lee, Suat Moi Puah

**Affiliations:** 1 Department of Biomedical Science, Faculty of Medicine, University of Malaya, Kuala Lumpur, Malaysia; 2 Faculty of Medicine, University of Malaya, University of Malaya, Kuala Lumpur, Malaysia; 3 Faculty of Science and Natural Resources, Universiti Malaysia Sabah, Jalan UMS, Kota Kinabalu, Sabah, Malaysia

**Keywords:** bimA, *Burkholderia pseudomallei*, Insulated isothermal PCR, On-site detection, Real-time PCR

## Abstract

**Background:**

* Burkholderia pseudomallei* causes melioidosis, a serious illness that can be fatal if untreated or misdiagnosed. Culture from clinical specimens remains the gold standard but has low diagnostic sensitivity.

**Method:**

In this study, we developed a rapid, sensitive and specific insulated isothermal Polymerase Chain Reaction (iiPCR) targeting *bimA* gene (*Burkholderia* Intracellular Motility A; BPSS1492) for the identification of *B. pseudomallei.* A pair of novel primers: BimA(F) and BimA(R) together with a probe were designed and 121 clinical *B. pseudomallei* strains obtained from numerous clinical sources and 10 ATCC non-targeted strains were tested with iiPCR and qPCR in parallel.

**Results:**

All 121 *B. pseudomallei* isolates were positive for qPCR while 118 isolates were positive for iiPCR, demonstrating satisfactory agreement (97.71%; 95% CI [93.45–99.53%]; *k* = 0.87). Sensitivity of the *bimA* iiPCR/POCKIT assay was 97.52% with the lower detection limit of 14 ng/µL of *B. pseudomallei* DNA*.* The developed iiPCR assay did not cross-react with 10 types of non-targeted strains, indicating good specificity.

**Conclusion:**

This *bimA* iiPCR/POCKIT assay will undoubtedly complement other methodologies used in the clinical laboratory for the rapid identification of this pathogen.

## Introduction

Melioidosis, a potentially fatal disease caused by the Gram-negative soil saprophyte *Burkholderia pseudomallei*, is found in soil and muddy water in endemic tropical areas, particularly in Southeast Asia and Northern Australia and can be acquired through inoculation or inhalation (Wiersinga et al., 2018). Inhalation of aerosolized bacteria can cause severe, rapid onset and fulminant disease while percutaneous inoculation is slower to progress and often limited to a cutaneous lesion. Mortality in septicemic melioidosis remains high as the bacteria are intrinsically resistant to many antimicrobial agents (Perumal Samy et al., 2017).

In Malaysia, the true incidence of melioidosis is not known, but cases have been reported in several states, including Kuala Lumpur (Puthucheary, 2009), Pahang (How et al., 2005), Johor (Pagalavan, 2005); Kedah (Hassan et al., 2010) and Kelantan (Zueter et al., 2018). Incidence vary between states and the highest was recorded in the largest rice producing state in Malaysia, i.e., Kedah with 16.35 per 100,000 population a year (Hassan et al., 2010). A recent review described the case fatality as ranging from one-third to about half of patients (33–54%) in four of the five Malaysian case reports (Nathan et al., 2018).

Thus far, culture remains the diagnostic gold standard where isolation of the bacterium from clinical specimens such as blood, urine, sputum or pus using selective agar requires 5 to 7 days (Chakravorty & Heath, 2019). However, culture-based methods have limited diagnostic sensitivities as *B. pseudomallei* can be misidentified as a contaminant or other species such as *B. cepacia*, *Bacillus* spp. or *Pseudomonas* spp. (Hoffmaster et al., 2015; Kingsley et al., 2016). Moreover, clinical diagnosis is often challenging and difficult as no specific pathognomonic features are evident (Dance, Limmathurotsakul & Currie, 2017). Delay in diagnosis and initiation of appropriate antimicrobial treatment can result in high mortality rates (Nathan et al., 2018).

Identification of *B. pseudomallei* could be difficult and confused with closely related species such as *B. mallei, B. thailandensis* and *B. cepacia* complex as variable results were seen in API 20NE biochemical test, latex agglutination test, Vitek 1 and Vitek 2 systems, Matrix-Assisted Laser Desorption Ionization-Time of Flight Mass Spectrometry (Dingle, Butler-Wu & Abbott, 2014; Lau et al., 2015; Songsri et al., 2018). An immunochromatographic lateral flow rapid diagnostic test, the Active Melioidosis Detect^TM^ has been developed by InBios (Seattle, USA) but showed an overall disappointing sensitivity and specificity (Rizzi et al., 2019).

To overcome these limitations, polymerase chain reaction (PCR)- based assays based on detection of nucleic acids of *B. pseudomallei* have been developed (Lau et al., 2015; Lowe et al., 2016). Traditional end-point PCR including monoplex, multiplex and nested PCR targeting specific regions of *B. pseudomallei* genome such as BPSS0658, TTS1, *mprA*, 16S rRNA. 23S rRNA and *fliC* have been evaluated for detecting *B. pseudomallei* DNA (Lau et al., 2015; Lowe et al., 2014; Meumann et al., 2006; Neubauer et al., 2007; Peddayelachagiri et al., 2018). Generally, these PCR assays demonstrated satisfactory specificities but sensitivities for diagnosis remain to be evaluated. Later, a more sensitive probe-based PCR such as TaqMan or SYBR green has been developed which gave quantitative information, called quantitative PCR (qPCR) (Lau et al., 2015; Lowe et al., 2014; Lowe 2016). Among qPCR studies, Novak et al.’s method (2006) targeted *orf 2* within TTS1 (present only in *B. pseudomallei*) and Bowers et al. (2010) targeted single nucleotide polymorphism in a conserved region that allows differentiation between *B. pseudomallei* and *B. mallei*. Both assays have shown promising accuracies in clinical specimens, but both traditional PCR and qPCR require sophisticated instruments and trained personnel (Lowe et al., 2014).

In contrast, isothermal amplification of nucleic acid enables rapid and efficient amplification at a constant temperature without the need for thermal cycling as required in PCR as well as a costly thermocycler (Zhao et al., 2015). Two different isothermal amplification techniques—loop-mediated isothermal amplification (LAMP) and recombinase polymerase amplification combined with lateral flow strip (LF-RPA) targeting the *orf2* of *B. pseudomallei*—have been developed (Chantratita et al., 2008; Peng et al., 2019). However, the LAMP assay had a diagnostic accuracy of 86.7% and is not sensitive when applied to blood samples (Chantratita et al., 2008). The LF-RPA assay can be visualized with a limit of detection as low as 20 femtogram (ca. 25.6 copies) but a few drawbacks were reported, including false positives in the non-template control and lack of internal controls. At present, rapid and better diagnostic methods are needed for more accurate detection of *B. pseudomallei*.

Recently, a low-cost convective PCR known as a fluorescent hydrolysis probe-based insulated isothermal PCR (iiPCR) has been reported. The amplification of short DNA or RNA fragments is carried out in a specially designed capillary tube, R-tube (GeneReach Biotechnology Corp., Taichung, Taiwan) with temperature gradient driven flows in a simple thermally baffled device (POCKIT™, GeneReach Biotechnology Corp., Taichung, Taiwan) in a relatively short period of time (Chang et al., 2012; Tsai et al., 2012). Good sensitivity and specificity of iiPCR has been reported for the detection of pathogens from clinical samples including malaria (Chua, Lee & Chai, 2016), dengue virus (Go et al., 2016; Tsai et al., 2018) and canine parvovirus (Wilkes et al., 2015) as well as seneca valley virus in swine from farms (Zhang et al., 2016) and white spot virus in shrimp (Tsai et al., 2012; Tsai et al., 2014). The significant progress development such as those highlighted above indicating the utility of this potential POCKIT™ system in the field to a wide range of pathogens including *B. pseudomallei.*

In the present study, considering all advantages of the iiPCR system, an iiPCR targeting the *Burkholderia* intracellular motility A (*BimA)* gene was developed for the identification of *B. pseudomallei* isolates*.* The *BimA* gene is involved in actin polymerization and annotated as BPSS1492 in the reference K96243 genome which located on the chromosome 2 of *B. pseudomallei* (Sitthidet et al., 2011). A high degree of *BimA* sequence conservation across 99 *B. pseudomallei* genomes of clinical or environmental origin from the area of endemicity has been reported, however, some orthologs are found in closely related virulent *B. mallei* ATCC23344 (BMAA0749) and the avirulent *B. thailandensis* E264 (BTH_II0875) (Sitthidet et al., 2008; Stevens et al., 2005). Interestingly, BimA protein of *B. pseudomallei* differ markedly in amino acid sequence at the N-terminus of the protein from the aforementioned species (Benanti, Nguyen & Welch, 2015; Stevens et al., 2005). In 2006, Ulrich and co-workers developed polymerase chain reaction (PCR) and qPCR targeting on *BimA* (BMAA0749) for the specify identification specification for *B. mallei* and differentiation from *B. pseudomallei* (Ulrich et al., 2006a; Ulrich et al., 2006b)*.* Hence, it is of interest to select *BimA* for *B. pseudomallei* identification, and the developed iiPCR could potentially be tested on various types of patient specimen in future.

## Material and Methods

### Bacterial genomic DNA

A total of 121 extracted genomic DNA from *B. pseudomallei* strains isolated from clinical specimens including blood, pus, swab, sputum, urine, and spenic biopsy, from patients at the University Hospital, University of Malaya, Kuala Lumpur (Puthucheary, 2009), were used for iiPCR and qPCR development.

Extracted DNA from *B. pseudomallei* K96243 was used for sensitivity test. A total of 10 commercially purchased strains (*non-Burkholderia pseudomallei*) were used for evaluating analytical specificities: *Aeromonas hydrophila* ATCC7966, *Aeromonas dhakensis* DSM17689, *Burkholderia cepacia* ATCC25416, *Burkholderia thailandensis* ATCC70038, *Escherichia coli* NCTC13476, *Pseudomonas putida* ATCC49128, *Pseudomonas aeruginosa* ATCC27853, *Pseudomonas fluorescens* ATCC13525, *Pseudomonas stutzeri* ATCC17588 and *Klebsiella pneumoniae* NCTC13443. All bacterial strains (non*-Burkholderia pseudomallei*) were revived from glycerol stock and grown on Luria–Bertani agar (Lennox) for 24 h at 37 °C. Total genomic DNA was prepared using boiling method (Puah et al., 2018). Briefly, 1.5 mL of the bacterial culture was pelleted, washed with deionized water and proceed to centrifugation. The isolated pellet was collected and resuspended with 100 µL deionized water. Bacterial suspension was incubated at 95° for 5 min and placed on ice immediately for 15 min. The crude lysate containing bacterial DNA was collected by centrifugation at 13,000 rpm for 5 min and transferred into new labelled tubes. The concentration and purity of DNA were measured by using NanoPhotometer (Implen, Germany) and standardized to 50 ng/µL for further investigation.

### Ethics statement

Approval from the University of Malaya Institutional Biosafety and Biosecurity Committee has been obtained for this study. The samples obtained are mainly used for diagnostic laboratory testing in the University Malaya Medical Centre, only the leftover and appropriate samples will be used in the research, hence each individual was verbally informed at the point of sample collection and this was sufficient at that material time.

### Design of primers and probes

Based on the 1,551 nucleotides (NC_006351.1:2033784–2035334), 100 global *B. pseudomallei* strains had 99.03–100% nucleotide identities. The primers targeting a highly conserved region in *BimA* gene of *B. pseudomallei* K96342 chromosome 2 was designed using Primer Express 3.0 (Applied Biosystems, CA, USA). Primers were designed to amplify a targert sequence length that less than 150 bp. Besides that, primer design with a length between 15 and 25 bp, melting temperature between 58 °C and 62 °C, GC content of the primer between 20 and 80%, avoid 3 G or C residues in a row near the 3′-end as well as avoid 4 or more repeated bases are the basic requirements for a POCKITT assay development. An oligonucleotide probe was designed to be detected by the fluorescent dye based on the recommended principles for iiPCR (Tsai et al., 2012; Chua, Lee & Chai, 2016). A BLAST search using Standard Nucleotide Basic Local Alignment Search Tool (BLASTN) (https://blast.ncbi.nlm.nih.gov/blast/Blast.cgi?PROGRAM=blastn&PAGE_TYPE=BlastSearch&LINK_LOC=blasthome) was performed on the resulting primers and probe against NCBI database to ensure their specificity to *B. pseudomallei* only.

### Testing of primers and TaqMan probe

Prior to qPCR development, the primers were tested using a conventional PCR. Ten µl of PCR reaction mix was prepared by mixing 1X MyTaq HS Mix (Bioline, London, UK), 10 µM of each BimA(F) and BimA(R) primers, and 50 ng of *B. pseudomallei* K96243 DNA. The reaction mix was subjected to PCR with initial denaturation at 95 °C for 1 min; 30 cycles of denaturation (95 °C for 30 s), annealing (55 °C for 30 s) and extension (72 °C for 10 s); and final extension at 72 °C for 1 min. The PCR products were electrophoresed on 1% (w/v) agarose gel pre-stained with safe-red and the DNA bands viewed using gel documentation unit (Major Science, CA, USA). The effectiveness of the probe was performed using qPCR.

### Development of *bimA* iiPCR

The reaction conditions using different combinations of concentration of primers and probe, the optimized assay was identified to consist of 2X MyTaq™ HS Mix (Bioline), 0.2 µM of BimA(F) and BimA(R) primers, 0.2 µM of BimA Probe and 1 µl of DNA sample in a total volume of 50 µl reaction mixture. The reaction mixture was transferred into an R-tube™ (GeneReach Biotechnology Corp., Taichung, Taiwan) and subjected to POCKIT™ Nucleic Acid Analyzer (GeneReach Biotechnology Corp., Taichung, Taiwan) with default thermal conditions. The reaction was completed in less than 1 h. The fluorescence signal to noise (S/N) ratio (signal_after_/signal_before_) measured at a wavelength of 520 nm (to detect FAM dye) were used as a threshold to determine positive or negative readouts: the result was displayed as “+” (positive: S/N >1.3), “ −” (negative: S/N < 1.2) or “?” (Undetermined: S/N 1.2–1.3). A “?” symbol indicates ambiguous result and the sample should be retested. The analytical sensitivity and detection limit of the iiPCR was evaluated using a ten-fold dilution series (10^0^ to 10^−5^) from genomic DNA of *B. pseudomallei* K96243, representing 140 ng, 14 ng, 1.4 ng, 0.14 ng, 0.014 ng, and 1.4 pg via three biological independent experiments. Specificity of the iiPCR assay was evaluated with DNA extracted from the 10 types of non-*Burkholderia pseudomallei* bacteria (ATCC strains).

### Reference *bimA* qPCR

To evaluate the performance of iiPCR in detecting *bimA* in *B. pseudomallei*, side-by-side comparison with that of the *bimA* qPCR was performed. A total volume of 10 µl qPCR reaction mixture containing 1X SensiFAST™ Probe Lo-ROX Master Mix (Bioline), 0.07 µM of BimA(F) and BimA(R) primers, 0.08 µM of BimA Probe and 50 ng of *B. pseudomallei* K96243 DNA samples was prepared and subjected to qPCR in Applied Biosystems 7500 Fast Real-Time PCR System. Following the manufacturer’s guidelines, the thermal cycling conditions were optimized as: polymerase activation at 95 °C for 2 min, 40 cycles of denaturation at 95 °C for 10 s and annealing/extension at 60 °C for 30 s. The *B. pseudomallei* K96243 was included in every run as positive control while water was used as non-template control. The analytical sensitivity of the qPCR was performed in three biological independent experiments and assays performed in triplicate at each dilution of *B. pseudomallei* K96243 DNA in single qPCR. A standard curve was generated by the LightCycler 4.0 software, slopes were used to calculate amplification efficiency and standard error while the detection limit of the assay was also evaluated. Standard curves were included in sample screening to determine the concentration of gDNA in the clinical sample by comparing the Ct’s. DNA extracted from 10 non-*B. pseudomallei* bacterial strains were used to assess the specificity of the assay by qPCR.

### Statistical analysis

Sensitivity and specificity of the iiPCR was calculated using MedCalc software. The degree of agreement iiPCR and qPCR assays was assessed by calculating Cohen’s kappa values (GraphPad Prism version 8.4.1).

## Results

### DNA extraction

The genomic DNA of *non-Burkholderia pseudomallei* were successfully extracted and the yield were in a range of 100–800 ng/uL with acceptable purity factor of 1.8 to 2.

### Primer design and probe

**Figure 1:**
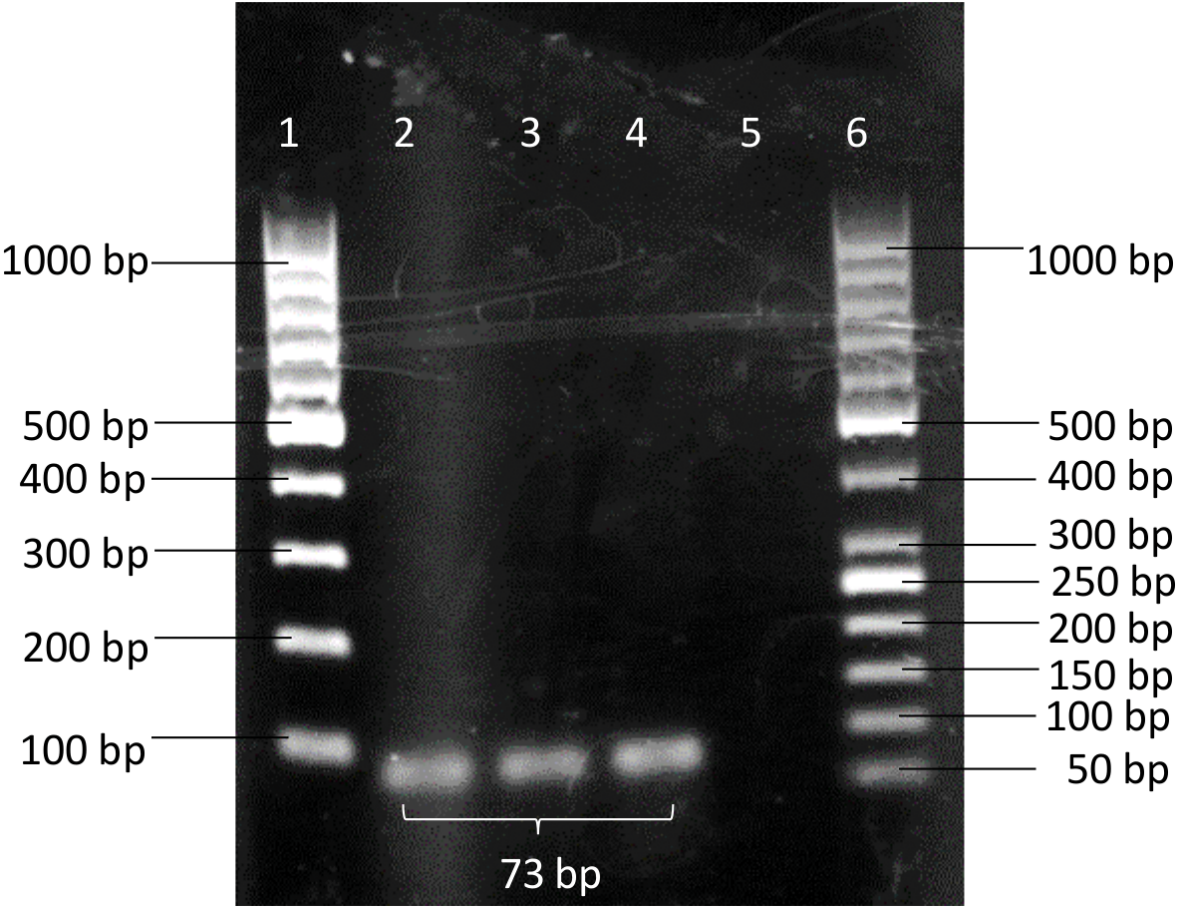
PCR products amplified using BimA(F) and BimA(R) primers. *B. pseudomallei* K96243 produced band with 73 bp. Lane 1–100 bp DNA ladder; Lanes 2 to 4 - *B. pseudomallei* **** K96243; Lane 5 - non-template control (NTC); Lane 6–50 bp DNA ladder.

A pair of novel primers: BimA(F) 5′-GTCTGCTGAAAACGCTCAATC-3′(positions 2034122 to 2034142) and BimA(R) 5′-TCGACTACGTCCTCGGTTACA-3′(positions 2034174 to 2034194) was designed from the *bimA* gene of *B. pseudomallei* K96342 chromosome 2 (GenBank accession no. NC_006351.1). A specific BimA probe 5′-CGGAGCTTCAGAACA- 3′(positions 2034152 to 2034166) was designed and labelled with a fluorescent reporter dye (FAM) at the 5′ end and TaqMan^®^ minor groove binder (MGB) with nonfluorescent quencher (NFQ) at the 3′ end. The primers were then checked through a BLASTN search and the results did not show any sequence match between primers and organisms other than *B. pseudomallei*. The conventional PCR showed the designed primer pairs BimA(F) and BimA(R) were able to amplify *B. pseudomallei* K96243 with an amplicon size of 73 bp (Fig. 1).

### Development of iiPCR

The analytical sensitivity of the iiPCR was evaluated using 10-fold serial dilutions of *B. pseudomallei* K96243 genomic DNA (3 biological replicates with known concentrations) (Table 1, Table S1). Positive signals were produced consistently from three independent experiments containing 14 ng/µL to 140 ng/µL of the gDNA with an average reading 1.447 ± 0.130 and 1.5743 ± 0.167, respectively, indicating that the BimA Probe-iiPCR assay was able to detect its target sensitively. However, no (0/3) positive signals were detected with S/N ratios of < 1.2 at 1.4 ng/µL and the subsequent 10-fold diluted DNA. Thus, the detection limit of *bimA* iiPCR was reached at 14 ng/µL. The developed iiPCR specifically reacted only with *B. pseudomallei* but did not cross-react with any of 10 non-*B. pseudomallei* bacteria by displaying “ −” signals with S/N ratios of < 1.2 (Table 1, Table S2).

### Reference *BimA* qPCR

**Figure 2:**
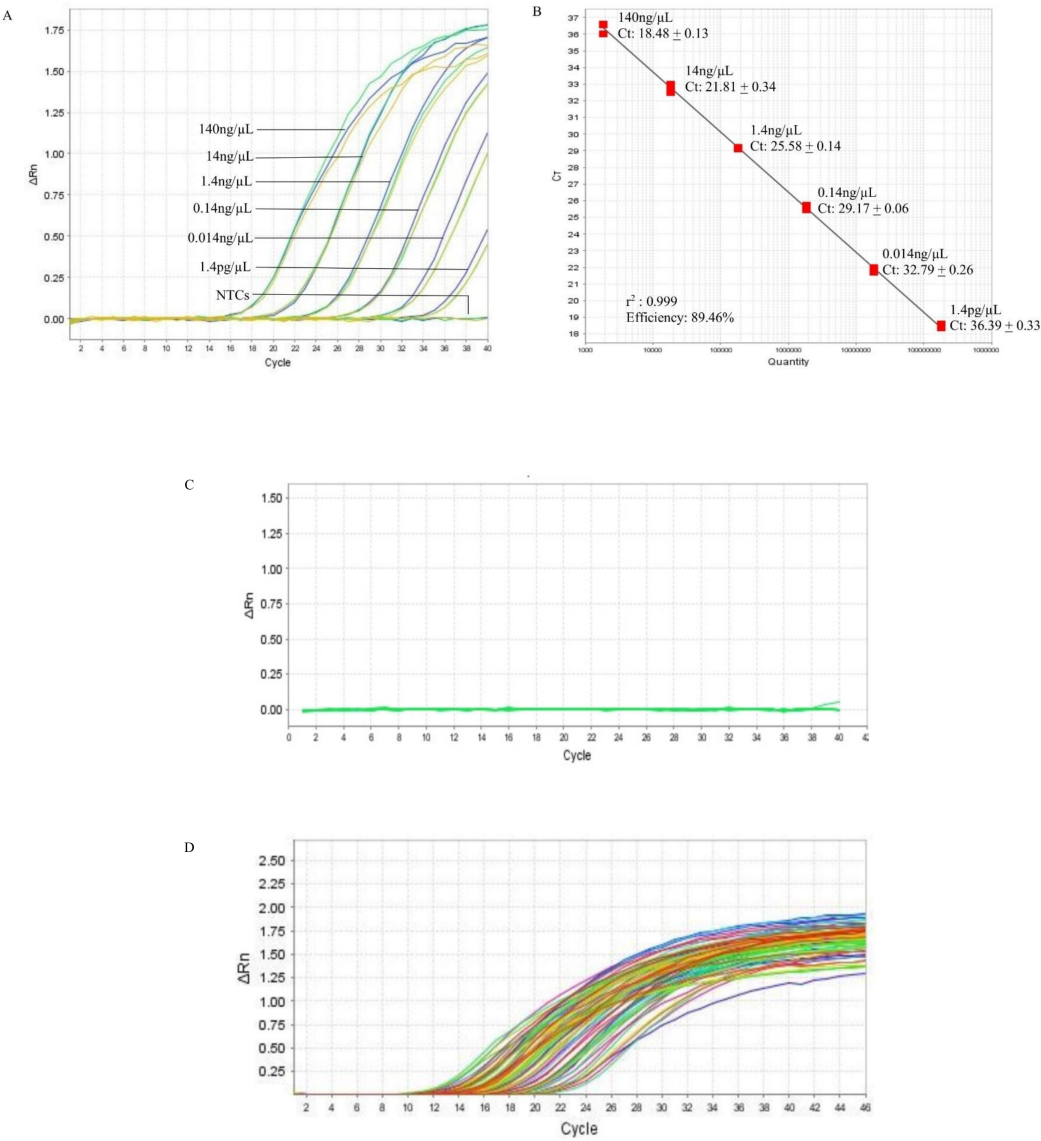
Reference *BimA* qPCR. (A) A representative of the amplification curves of the 10-fold serial diluted DNA of *B. pseudomallei* K96243 that consisted of three data points for each dilution point, i.e., 140 ng, 14 ng, 1.4 ng, 0.14 ng, 0.014 ng, and 1.4 pg. The detection limit of the assay is 1.4 pg /µL and NTCs (non-template controls) did not produce curves. (B) A representative standard curve constructed with 10-fold serial diluted DNA of *B. pseudomallei* K96243. The efficiency of the assay was 89.46%, with r-squared value of 0.999 for the triplicates. The standard curved is consisted of three data points for each 10X DNA dilution point in a single qPCR. (C) Amplification plot of the 10 non-*B. pseudomallei* bacteria using 50 ng/µL of their genomic DNA in the specificity test. No non-*B. pseudomallei* bacteria was detected by the assay (green lines). (D) Amplification plot shows the amplification curves of some of the *B. pseudomallei* clinical samples with Ct values ranging from 9.91 to 36.54 that correspond to 5,560 ng and 7.2 pg of DNA respectively (colored lines). All clinical samples could be detected by the assay.

In the qPCR, the standard curve indicated that the detection limit of the assay could be down to 1.4 pg/µL (Fig. 2A, Table S3). The r^2^ and the efficiency of the assay was 0.999 and 89.46%, respectively (Fig. 2B). The qPCR of the 10 non-*B. pseudomallei* bacterial strains did not produce amplification curves (Fig. 2C, Table S2) indicating that the qPCR assay was 100% specific to *B. pseudomallei.* The screening of the 121 *B. pseudomallei* clinical samples all gave positive results, producing amplification curves with Ct’s ranging from 9.91 to 36.54 that correspond to 5560 ng and 7.2 pg of DNA (Fig. 2D, Table S4). Hence the sensitivity of the qPCR assay in *B. pseudomallei* samples was 100%.

**Table 1:** Analytical sensitivity and specificity of iiPCR and qPCR for detection of *Burkholderia pseudomallei*.

**Analysis**	**Organism**	**Dilution**	**DNA concentration**	**Detection results**			
				**iipcr**	**Average of S/N ratios** (± **SD)**	**qPCR(Ct)**	**Average of Ct’s value** (± **SD)**
Sensitivity	*Burkholderia pseudomallei* K96243	10	140 ng/µL	+ + +	1.573 ± 0.167	18.48 16.94 16.60	17.34 ± 0.82
		10^−1^	14 ng/µL	+ + +	1.447 ± 0.130	21.81 20.91 20.40	21.04 ± 0.58
		10^−2^	1.4 ng/µL	- - -	1.133 ± 0.021	25.58 24.94 24.66	25.06 ± 0.39
		10^−3^	14pg/µL	- - -	1.056 ± 0.033	29.17 29.01 28.77	28.98 ± 0.16
		10^−4^	1.4pg/µL	- - -	1.025 ± 0.005	32.79 32.93 32.71	32.81 ± 0.09
		10^−5^	1.4pg/µL	- - -	1.034 ± 0.021	36.39 36.81 36.52	36.57 ± 0.18
		Water	0 fg/µL	- - -	1.016 ± 0.026	No detected	
Specificity	*Aeromonas hydrophila* ATCC7966			–	1.0465	No detected	
	*Aeromonas caviae* ATCC15468			–	1.1969	No detected	
	*Burkholderia cepacia* ATCC25416			–	1.0376	No detected	
	*Burkholderia thailandensis* ATCC70038			–	1.1346	No detected	
	*Escherichia coli* NCTC13476			–	1.0354	No detected	
	*Pseudomonas putida* ATCC49128			–	1.1586	No detected	
	*Pseudomonas aeruginosa* ATCC27853			–	1.1827	No detected	
	*Pseudomonas fluorescens* ATCC13525			–	1.0795	No detected	
	*Pseudomonas stutzeri* ATCC17588			–	1.1183	No detected	
	*Klebsiella pneumoniae* NCTC13443			–	1.0362	No detected	

**Notes.**

Sensitivity were tested in triplicate, and one replicate for specify test.

### Comparison of analytical sensitivity, specificity, and performance of iiPCR with qPCR

The same genomic DNA (*B. pseudomallei* and non-*B. pseudomallei*) was tested by iiPCR and qPCR in parallel with results shown in Table 2. Both iiPCR and qPCR generated negative S/N signals or no amplification curves with all the non-*B. pseudomallei* tested, demonstrating excellent specificity for *B. pseudomallei*. Of the 121 isolates that were positive for *B. pseudomallei*, qPCR showed 100% sensitivity but only 96.72% (118/121) sensitivity for iiPCR. Four iiPCR ambiguous samples (78, 114, 115 and 145) showed signals “?” with S/N ratio of 1.2464, 1.2610, 1.2716 and 1.2655 (Table S4). After repeating the test once, two samples 114 and 115 demonstrated signal “-” while sample 78 and 145 showed signal “+” (Table 3). Of 10 non-*B. pseudomallei* samples, both iiPCR and qPCR showed 100% specificity. In summary, the agreement between iiPCR and qPCR assay was about 97.71% (95% confidence interval, 93.45 to 99.53%) with a kappa value of 0.87 (Table 2).

**Table 2:** Agreement between iiPCR and qPCR for *Burkholderia pseudomallei* detection.

**iiPCR**	**qPCR**		**Total**
	**Positive (%)**	**Negative (%)**	
Positive	118 (97.5%)	0	118 (90.1%)
Negative	3 ( 2.5%)	10 (100%)	13 ( 9.9%)
Total	121	10	131

**Notes.**

Agreement (95% CI) –97.71% (93.45% to 99.53%).

Kappa = 0.857.

**Table 3:** Clinical samples tested with the iiPCR showing undetermined (?) detection and analysed in parallel with the qPCR assay.

**Sample**	**DNA concentration^a^**	**iiPCR**			**qPCR**	
		**S/N ratio**			**Ct value**	**Interpretation**
		**First reading**	**Second reading**	**Interpretation**		
78	10.65 ng/µL	1.2464	1.3259	? +	21.96	Positive
114	12.18 ng/µL	1.2610	1.1447	? -	22.50	Positive
115	10.16 ng/µL	1.2716	1.1841	? -	22.84	Positive
145	12.37 ng/µL	1.2655	1.8492	? +	22.33	Positive

**Notes.**

a The DNA used are based on 1 µL volume to mimic the actual scenario of a rapid test where a fixed protocol has been applied for the assay.

## Discussion

Diagnosis of melioidosis is challenging for clinicians due to non-specific and protean manifestations of the disease ranging from localized abscess formation to disseminated abscesses and septicemia (Puthucheary, 2009; Chakravorty & Heath, 2019). The burden of disease-associated mortality is high and cases are increasingly being reported in other parts of the world besides the endemic regions–northeast Thailand, Malaysia, Singapore, and northern Australia (Perumal Samy et al., 2017).

In this study, the *bimA* iiPCR/POCKIT assay targeting the *bimA* gene of *B. pseudomallei* was developed to facilitate disease management and surveillance to enable early intervention. The *bimA* iiPCR/POCKIT system offers qualitative PCR results with minimal hands-on steps and discrepancies in interpretation of results can be avoided.

The developed *bimA* iiPCR/POCKIT assay was analyzed by the reference qPCR in parallel. Our findings demonstrated that both iiPCR and qPCR assays are specific and do not cross-react with other non-*B. pseudomallei* pathogens (Table 1). A highly conserved nucleotide region within *bimA* gene of 100 global *B. pseudomallei* strains was found to be located from position 2033784 to 2034605 (52.9%, 821/1551). Then, the forward and reverse primers as well as probes were designed within this 821-nucleotides target region. The *in silico* BLASTN analysis revealed that primers are high specificity and it does not cross-react with non-*B. pseudomallei* pathogens including closely related *Burkholderiaceae*. However, one limitation in this study is that limited number of isolates from *Burkholderiaceae* was investigated. Thus, more isolates from *Burkholderia* genus particularly *B. mallei* and *B. thaialandensis* should be included in future study to further substantiate the results.

Our results in this study present the stability of the target *bimA* sequence the stability to specifically detect 118 of 121 *B. pseudomallei* clinical strains. As demonstrated in the analytical sensitivity test in Table 1, iiPCR and qPCR were able to detect 14 ng/µL *B. pseudomallei* K96243 DNA, so 100% agreement was found between both assays for 98 samples consisting of *B. pseudomallei* DNA concentrations between 15.1 to 5560.8 ng/µL. Another 23 samples containing DNA below 14 ng/µL (ranging from 7.2 pg/µL to 13.65 ng/µL) were found to be positively detected using qPCR as the assay was more sensitive where the DNA can go down to 1.4 pg/µL with high Ct values. These 23 samples were anticipated to be negatively detected for iiPCR as DNA concentrations are below the detection limit for this method (below 14 ng/µL). However, 18 (78%) samples tested positive with S/N 1.3496 to 2.2389, 1 (4%) sample tested negative with S/N 1.1718 and 4 (17%) samples - 78, 114, 115 and 145 showed “?”. After repeating the test, 2 samples (78 and 145) tested positive whereas another 2 samples (114 and 115) tested negative. This indicates that the assay could possibly detect samples below the lower of detection limit. Overall, iiPCR showed satisfactory agreement with the qPCR to detect *B. pseudomallei* (97.71%; 95% CI [93.45–99.53%]; Cohen’s kappa value = 0.857; *n* = 131).

Overall, the developed *bimA* iiPCR/POCKIT assay in this study is useful and suitable to aid in rapid identification of *B. pseudomallei* although not sensitive and quantitative as the qPCR method. The iiPCR assay successfully amplified the target DNA within *bimA* using crude genomic DNA lysates of *B. pseudomallei*, which suggests isothermal nucleic acid amplification methods can shorten the time for *B. pseudomallei* identification compared to culture-based isolation*.* However, it should be noted that various yields of genomic DNA were obtained using similar volume of bacterial culture through a simple boiling method. Differences in the amount of DNA released is largely dependent on cell lysis from bacterial suspension. One of the possible explanations is due to the efficacy of mechanical disruption of bacterial pellet using pipetting for the preparation of homogenized suspension. Therefore, this step should be conducted in more carefully in future study.

This iiPCR method may overcome some limitations of cultural diagnosis as some cultures may resemble contaminants and be discarded erroneously due to samples collected from non-sterile sites from suspected melioidosis patients (Hoffmaster et al., 2015). Hence, this *bimA* iiPCR/POCKIT assay can serve as an alternative economic approach for *B. pseudomallei* identification and differentiation for other closely related *Burkholderiacea*. Low cost POCKIT™  Nucleic Acid Analyzer device is capable of generating results for up to 8 nucleic acid samples within 1 h. A higher throughput of 64 samples per 8 h workday is also feasible. For potential field deployment, the POCKIT™  Nucleic Acid Analyzer device can utilize power from a car battery or a rechargeable battery. Previous studies reported that the bacterial load in clinical samples can vary greatly based on the type of specimens (Wongsuvan et al., 2009). Tellapragada et al. (2017) described that *B. pseudomallei* has the highest microbial load from sputum and pus specimens and particularly low in blood. Therefore, future studies on testing DNA extracted directly from clinical specimens is required to evaluate the feasibility and performance of this instrument for on-site detection of *B. pseudomallei*.

## Conclusion

This *bimA* POCKITT/iiPCR assay will undoubtedly complement other methodologies used in the clinical laboratory for the rapid identification of this pathogen.

##  Supplemental Information

10.7717/peerj.9238/supp-1 Table S1 Sensitivity of iiPCR

10.7717/peerj.9238/supp-2 Table S2 Specificity of both qPCR and iiPCR

10.7717/peerj.9238/supp-3 Table S3 Sensitivity of qPCR

10.7717/peerj.9238/supp-4 Table S4 Screening results using qPCR and iiPCR

10.7717/peerj.9238/supp-5 Supplemental Information 5 Tables S1 - S4

## Additional Information and Declarations

### Data Availability

The following information was supplied regarding data availability: The data is available in the Methods section and Tables S1–S4.
