# Supplementary material for: Rapid identification of melioidosis agent by an insulated isothermal PCR on a field–deployable device
Source: PeerJ. 2020 May 27;8:e9238. doi: 10.7717/peerj.9238 (PMC7261116; doi:10.7717/peerj.9238)
Supplement: Supplemental Information 5 [file peerj-08-9238-s005.pdf]

Table S1: Sensitivity of iiPCR

First Experiment	Sample	Positive Control	Negative (Water)	140 ng	14 ng	1.4 ng	0.14 ng	0.014 ng	0.0014 ng
	Signal	(+)	(-)	(+)	(-)	(-)	(-)	(-)	(-)
	R1	1.3355	0.988	1.3423	1.1158	1.0122	1.0315	1.0048	1.0203

Second Experiment	Sample	Positive Control	Negative (Water)	140 ng	14 ng	1.4 ng	0.14 ng	0.014 ng	0.0014 ng
	Signal	(+)	(-)	(+)	(-)	(-)	(-)	(-)	(-)
	R2	1.3756	1.0077	1.6521	1.1203	1.0916	1.0188	1.0466	1.0397

Third experiment	Sample	Positive Control	Negative (Water)	140 ng	14 ng	1.4 ng	0.14 ng	0.014 ng	0.0014 ng
	Signal	(+)	(-)	(+)	(-)	(-)	(-)	(-)	(-)
	R3	1.6301	1.0508	1.7285	1.1616	1.0639	1.0261	1.0507	1.0314

Table S2: Specificity of both qPCR and iiPCR

Organisms	qPCR	iiPCR	
		Reading	Interpretation
Aeromonas hydrophila ATCC7966	Not detected	1.0465	(-)
Aeromonas caviae ATCC15468	Not detected	1.1969	(-)
Burkholderia cepacia ATCC25416	Not detected	1.0736	(-)
Burkholderia thailandensis ATCC70038	Not detected	1.1346	(-)
Escherichia coli NCTC13476	Not detected	1.0354	(-)
Pseudomonas putida ATCC49128	Not detected	1.1586	(-)
Pseudomonas aeruginosa ATCC27853	Not detected	1.1827	(-)
Pseudomonas fluorescens ATCC13525	Not detected	1.0795	(-)
Pseudomonas stutzeri ATCC17588	Not detected	1.1183	(-)
Klebsiella pneumoniae NCTC13443	Not detected	1.0362	(-)

Table S3: Sensitivity of qPCR

Exp	DNA Concentration	Replicate 1	Replicate 2	Replicate 3	Average
	140 ng	18.62	18.42	18.39	18.48
	14 ng	21.76	21.7	21.97	21.81
	1.4 ng	25.72	25.56	25.45	25.58
1	0.14 ng	29.21	29.2	29.11	29.17
	0.014 ng	32.97	32.92	32.49	32.79
	0.0014 ng	36.56	36.59	36.01	36.39
	NTC	Not detected	Not detected	Not detected	-
	140 ng	16.8	17.01	17.02	16.94
	14 ng	20.39	21.46	20.89	20.91
	1.4 ng	24.93	24.88	25.02	24.94
2	0.14 ng	29.16	28.95	29.12	29.01
	0.014 ng	33.06	32.93	32.81	32.93
	0.0014 ng	36.85	36.9	36.67	36.81
	NTC	Not detected	Not detected	Not detected	-
	140 ng	16.53	16.62	16.65	16.6
	14 ng	20.35	20.54	20.3	20.4
	1.4 ng	24.69	24.67	24.62	24.66
3	0.14 ng	28.68	28.68	28.93	28.77
	0.014 ng	32.97	32.42	32.74	32.71
	0.0014 ng	36.17	36.43	36.95	36.52
	NTC	Not detected	36.91	Not detected	-

Table S4: Screening results using qPCR and iiPCR

No.	DNA Sample	DNA concentration (ng/uL)	qPCR Ct	POCKIT Run 1 Reading	POCKIT Run 1 Interpretation	POCKIT Repeated run Reading	POCKIT Repeated run Interpretation
1	Sample 1	51.43	18.93	1.9258	(+)		
2	Sample 2	99.83	17.65	1.6649	(+)		
3	Sample 3	463.12	14.69	2.1227	(+)		
4	Sample 4	12.38	21.67	1.4625	(+)		
5	Sample 5	63.29	18.53	1.8725	(+)		
6	Sample 6	528.87	14.44	2.3061	(+)		
7	Sample 7	28.15	20.09	1.6013	(+)		
8	Sample 8	77.79	18.13	1.7079	(+)		
9	Sample 9	978.93	13.25	1.9092	(+)		
10	Sample 10	257.43	15.82	1.3542	(+)		
11	Sample 11	1557.85	12.36	1.7717	(+)		
12	Sample 12	529.34	14.44	1.7657	(+)		
13	Sample 13	206.97	16.24	1.8556	(+)		
14	Sample 14	511.79	14.5	2.0848	(+)		
15	Sample 15	459.35	14.71	1.8401	(+)		
16	Sample 16	1377.57	12.59	1.8103	(+)		
17	Sample 17	345.03	15.26	2.0142	(+)		
18	Sample 18	87.35	17.91	1.9794	(+)		
19	Sample 19	191.81	16.39	1.7607	(+)		
20	Sample 20	1744.53	12.14	2.1174	(+)		
21	Sample 21	374.84	15.1	2.3166	(+)		
22	Sample 22	791.48	13.66	1.8189	(+)		
23	Sample 23	763.66	13.73	1.7449	(+)		
24	Sample 24	387.1	15.04	2.0983	(+)		
25	Sample 25	132.68	17.1	1.8367	(+)		
26	Sample 26	284.29	15.63	1.6234	(+)		
27	Sample 27	39.25	20.3	1.676	(+)		
28	Sample 28	22.84	20.49	1.7285	(+)		
29	Sample 29	5560.81	9.91	2.2635	(+)		
30	Sample 30	549.9	14.36	1.7902	(+)		
31	Sample 31	166.38	16.66	1.7409	(+)		
32	Sample 32	15.1	21.29	1.7228	(+)		
33	Sample 33	37.38	19.54	1.8173	(+)		
34	Sample 34	354.77	15.21	1.9319	(+)		
35	Sample 35	198.21	16.33	2.1163	(+)		
36	Sample 36	35.38	19.65	1.5086	(+)		
37	Sample 37	178.32	16.53	2.1671	(+)		
38	Sample 38	37.39	19.54	1.6747	(+)		
39	Sample 40	307.85	15.48	2.1603	(+)		
40	Sample 41	294.54	15.57	2.1217	(+)		
41	Sample 42	624.02	14.12	2.3712	(+)		
42	Sample 43	768.1	13.72	2.7875	(+)		

No.	DNA Sample	DNA concentration (ng/uL)	qPCR	POCKIT			
				Run 1		Repeated run	
			Ct	Reading	Interpretation	Reading	Interpretation
43	Sample 44	383.79	15.06	1.904	(+)		
44	Sample 45	5.72	23.16	1.6688	(+)		
45	Sample 46	980.62	14.23	2.1653	(+)		
46	Sample 47	180.53	16.51	1.9493	(+)		
47	Sample 48	95.76	17.73	1.7781	(+)		
48	Sample 49	621.35	14.13	1.9796	(+)		
49	Sample 50	241.62	15.95	2.0542	(+)		
50	Sample 51	188.54	16.42	1.8819	(+)		
51	Sample 52	139.06	17.01	1.7213	(+)		
52	Sample 54	97.21	17.7	1.7835	(+)		
53	Sample 55	537.22	14.41	1.8797	(+)		
54	Sample 56	154.34	16.81	1.7307	(+)		
55	Sample 57	2266.3	11.64	2.2382	(+)		
56	Sample 58	7.44	22.65	1.6744	(+)		
57	Sample 59	586.53	14.24	2.0138	(+)		
58	Sample 60	225.4	16.08	2.2473	(+)		
59	Sample 61	182.55	16.49	1.7981	(+)		
60	Sample 62	32.96	19.78	1.926	(+)		
61	Sample 63	38.93	19.46	2.1981	(+)		
62	Sample 64	52.9	18.87	2.2395	(+)		
63	Sample 65	83.04	18	2.1377	(+)		
64	Sample 68	57.56	18.71	2.3825	(+)		
65	Sample 69	9.55	22.17	2.2389	(+)		
66	Sample 70	30.13	19.96	1.5696	(+)		
67	Sample 71	33.18	19.77	1.8973	(+)		
68	Sample 72	33.96	19.72	2.0959	(+)		
69	Sample 73	159.45	16.75	2.0235	(+)		
70	Sample 74	30.43	19.94	1.7799	(+)		
71	Sample 75	31.41	19.88	1.8843	(+)		
72	Sample 76	21.66	20.59	2.1206	(+)		
73	Sample 77	303.37	15.51	2.0786	(+)		
74	Sample 78	10.65	21.96	1.2464	(?)	1.3259	(+)
75	Sample 79	1661.55	12.23	2.0802	(+)		
76	Sample 80	201.12	16.3	2.4792	(+)		
77	Sample 81	1141.7	12.96	2.2852	(+)		
78	Sample 82	318.03	15.42	2.4204	(+)		
79	Sample 83	796.69	13.65	1.4271	(+)		
80	Sample 84	1392.17	12.57	1.3731	(+)		
81	Sample 86	328.03	15.36	1.397	(+)		
82	Sample 87	676.7	13.96	1.3713	(+)		
83	Sample 88	704.63	13.89	1.5942	(+)		
84	Sample 89	0.0097	35.44	1.4461	(+)		
85	Sample 90	1139.22	12.96	2.1638	(+)		
86	Sample 91	10.92	21.91	2.1512	(+)		

No.	DNA Sample	DNA concentration (ng/uL)	qPCR	POCKIT			
				Run 1		Repeated run	
			Ct	Reading	Interpretation	Reading	Interpretation
87	Sample 92	219.09	17.05	1.9196	(+)		
88	Sample 93	2127.95	12.76	1.9845	(+)		
89	Sample 94	160.25	17.64	1.6003	(+)		
90	Sample 96	252.12	16.79	2.2921	(+)		
91	Sample 97	189.32	17.33	2.0566	(+)		
92	Sample 98	5.9	23.87	1.3995	(+)		
93	Sample 99	113.84	18.29	1.7749	(+)		
94	Sample 100	379.62	16.02	2.0418	(+)		
95	Sample 101	1165.03	13.9	2.204	(+)		
96	Sample 102	83.58	18.87	1.9668	(+)		
97	Sample 103	120.01	18.19	2.1561	(+)		
98	Sample 104	41.31	20.2	1.6242	(+)		
99	Sample 105	529.66	15.39	2.3061	(+)		
100	Sample 106	47.59	19.93	2.1752	(+)		
101	Sample 107	7.97	23.3	1.67	(+)		
102	Sample 108	10.44	22.79	1.372	(+)		
103	Sample 109	352.33	16.16	2.3125	(+)		
104	Sample 110	459.9	15.65	1.4939	(+)		
105	Sample 111	13.65	22.29	1.3872	(+)		
106	Sample 112	19.26	21.64	1.374	(+)		
107	Sample 114	12.18	22.5	1.261	(?)	1.1447	(-)
108	Sample 115	10.16	22.84	1.2716	(?)	1.1841	(-)
109	Sample 117	6.54	23.68	1.395	(+)		
110	Sample 118	11.31	22.64	1.4136	(+)		
111	Sample 119	2.36	25.6	1.5318	(+)		
112	Sample 121	13.12	22.36	1.3496	(+)		
113	Sample 122	20.43	21.53	1.7278	(+)		
114	Sample 123	7.05	23.53	1.768	(+)		
115	Sample 124	8.54	23.17	1.6229	(+)		
116	Sample 126	19.32	21.63	1.3567	(+)		
117	Sample 127	0.0118	35.59	1.3645	(+)		
118	Sample 129	0.0072	36.54	1.1718	(-)		
119	Sample 130	41.71	20.18	1.6814	(+)		
120	Sample 139	7.82	23.34	1.4622	(+)		
121	Sample 145	13.37	22.33	1.2655	(?)	1.8492	(+)
